# Development, Characterizations and Biocompatibility Evaluations of Intravitreal Lipid Implants

**DOI:** 10.17795/jjnpp-16414

**Published:** 2014-04-07

**Authors:** Lana Tamaddon, Abolfazl Mostafavi, Mohammad Riazi-esfahani, Reza Karkhane, Sara Aghazadeh, Morteza Rafiee-Tehrani, Farid Abedin Dorkoosh, Fahimeh Asadi Amoli

**Affiliations:** 1 Department of Pharmaceutics, Isfahan Pharmaceutical Sciences Research Center, School of Pharmacy and Pharmaceutical Sciences, Isfahan University of Medical Sciences, Isfahan, IR Iran; 2 Eye Research Center, Farabi Eye Hospital, Tehran University of Medical Sciences, Tehran, IR Iran; 3 Department of Pharmaceutics, Faculty of Pharmacy, Tehran University of Medical Sciences, Tehran, IR Iran

**Keywords:** Triglyceride, Disease, Toxicology

## Abstract

**Background:**

The treatment of posterior eye diseases is always challenging mainly due to inaccessibility of the region. Many drugs are currently delivered by repeated intraocular injections.

**Objectives:**

The purpose of this study was to investigate the potential applications of natural triglycerides as alternative carriers to synthetic polymers in terms of drug release profile and also biocompatibility for intraocular use.

**Materials and Methods:**

*In vitro*/*in vivo* evaluations of intravitreal implants fabricated from the physiological lipid, glyceride tripalmitate containing clindamycin phosphate as a model drug was performed. The micro-implants with average diameter of 0.4 mm were fabricated via a hot melt extrusion method. The extrudates were analyzed using scanning electron microscopy, differential scanning calorimetry, and *in vitro* drug dissolution studies. For biocompatibility, the implants were implanted into rabbit eyes. Clinical investigations including fundus observations, electroretinography as well as histological evaluations were performed.

**Results:**

*In vitro* tests guaranteed usefulness of the production method for preparing the homogenous mixture of the drug and lipid without affecting thermal and crystalinity characteristics of the components. *In vitro* releases indicated a bi-phasic pattern for lower lipid ratios, which were completed by the end of day three. With higher lipid ratios, more controlled release profiles were achieved until about ten days for a lipid ratio of 95%. Clinical observations did not show any abnormalities up to two months after implantation into the rabbit eye.

**Conclusions:**

These results suggest that although the implant could not adequately retard release of the present drug model yet, due to good physical characteristics and *in vivo* biocompatibility, it can represent a suitable device for loading wide ranges of therapeutics in treatment of many kinds of retinochoroidal disorders.

## 1. Background

The treatment of posterior eye diseases is always challenging mainly due to the inaccessibility of the region. Many drugs are currently delivered by repeated intraocular injections. To avoid the risks and serious complications associated with this method, several implantable controlled-release devices have been investigated for delivering the drug directly to affected areas (1). Intraocular implants, fabricated from different materials, have been evaluated as sustained drug delivery systems. The main advantage of solid implants is avoidance of clarity impairment of the vitreous media compared to particulate drug delivery systems (1).

The first intraocular implants were non-biodegreadable; Vitasert^©^, Retisert^©^ and Medidur^©^ are examples of these devices. Despite many advantages, there are some limitations with the application of these systems, such as the need for surgery for implantation which can be associated with serious complications such as endophthalmitis. Also, another surgical procedure is required for their removal (1). Recently, intraocular implants of biodegradable polymers for controlled drug delivery in the vitreous have been investigated. Polymers such as poly lactic acid (PLA) and polylactic-glycolic acid (PLGA), have recently been used to produce intravitreal implants (2, 3). Biodegradable polymer implants have some advantages over their non-biodegradable counterparts. They do not need to be removed once the drug is depleted due to conversion to a soluble form through body reactions. Since, these devices can be formed into various shapes, they offer the potential to be implanted by injection with a simple procedure. This way, common complications associated with non-biodegradable implants could be minimized (2, 3). Natural lipids, e.g. triglycerides and cholesterol, may have the potential to be used as biocompatible and biodegradable carriers for many of the therapeutics (4). Recently, the evaluation of these natural biocompatible materials as substitutes for synthetic polymers in controlled release systems has drawn much interest. Using these materials, disadvantages of their synthetic counterparts can be avoided (5). With these materials no swelling occurs (6) and the formation of acidic degradation products, which can result from biodegradation of some synthetic polymers like PLA and PLGA (7), are not of concern. In this project, we focused on the manufacture of miniaturized triglyceride implants. Glyceryl tripalmitate (GTP) was selected as a matrix former because its good compressibility allows its formation into an implant and also its capability of long-term drug releases. The biocompatibility of GTP implants in some parts of the body was previously approved (8-10). These properties suggest that GTP can serve as an alternative to polymeric release systems.

The purpose of this approach was to evaluate, for the first time, the the drug release profile and also the ocular tolerance of the new intravitreal lipid implants in rabbit eyes. This novel device was compared with the PLA polymer, which is considered to be well tolerated by retinochoroidal tissues. Clindamycin phosphate (CLP) served as a model compound for release experiments, as it is highly water soluble and therefore, a good indicator for the ability of the matrices to control release. Sustained release intraocular implants loaded with CLP were formerly fabricated by our group in the pharmaceutical laboratory of Isfahan University of Medical Sciences, using PLA. The results showed promising results with controlled release of CLP for about five weeks.

## 2. Objectives

In the present study, a sustained release device made of GTP as a natural triglyceride, was evaluated in terms of its drug release profile and *in vivo* tolerance in rabbit eyes.

## 3. Materials and Methods

### 3.1. Materials

Glyceryl tripalmitate was obtained from TCI (Tokyo, Japan). Clindamycin phosphate was kindly donated by Behdaroo Co. (Tehran, IR Iran). PLA R203 (molecular weight 18 kDa) was purchased from Sigma Aldrich (Germany). All other chemicals were obtained from Merck (Darmstadt, Germany).

### 3.2. Preparation of the Implants

**Figure 1.**
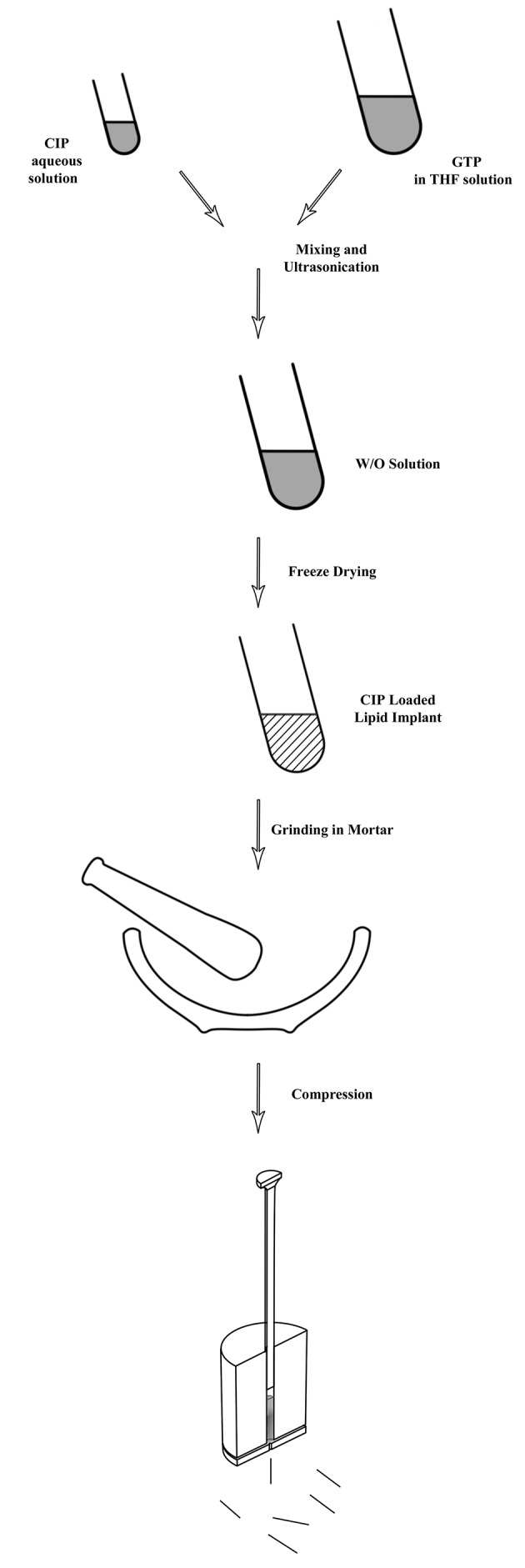
Schematic Representation of the Manufacturing Procedure for Lipid Implants

For the aseptic production of the matrices, GTP was tempered for two hours at 160˚C. Next, crystallization of the lipid in the stable beta-orientation was achieved by heating the molten lipid for three days at 55˚C (11). CLP loaded lipid implants were prepared in a two-step process. Figure 1 shows schematic steps of the preparation method. In the first step, homogenous mixtures of different GTP to CLP ratios (50-95%) were prepared via an emulsion technique. In this method, firstly, constant amounts of CLP were dissolved in water. After filtration through 0.22 µm, the solution was dispersed in another solution containing different amounts of the GTP in tetrahydrofurane (THF) under vigorous vortex mixing.

The resulting mixture was sonicated for 30 seconds at a frequency of 20 kHz and an intensity of 120 Watts (Bandelin, Germany). After obtaining W/O emulsion, the mixture was placed under liquid nitrogen until the THF was completely frozen. The organic solvent was removed by applying a vacuum in the freeze dryer (chemical free freeze dryer, Operon, Gyenggi, Korea), for 24 hours under aseptic conditions, leaving behind a powder formulation. The powder was collected and ground in a mortar. For manufacturing of the lipid implants, a hot melt extrusion method using a laboratory scale vertical ram extruder equipped with a set of 2 mm diameter cylindrical punches and a die plate with 23-gauge of 0.4 mm diameter was used. The powder was fed into the barrel of the extruder and was extruded at 30˚C by applying manual compression with approximately 139 bar pressure. Extrudates with average diameters of 0.41 mm were obtained. The average weight of extrudates was 5.3 mg ± 0.5 mg. Placebo lipid implants were prepared by using the above mentioned method after introducing ground lipid powder inside the extruder.

### 3.3. In vitro Release Studies

*In vitro* release studies were conducted under sink conditions over 1 week. Briefly, three implants were incubated inside three different vials containing 1 ml of phosphate buffer (pH = 7.4). The tubes were placed inside a batch shaker at 37˚C ± 0.5˚C. The samples were shaken at 50 rpm. At different time points, the medium was completely collected and replaced immediately by 1.0 mL of fresh buffer. Collected samples were centrifuged at 5000 rpm for 10 minutes, filtered and stored in a refrigerator. The amount of clindamycin released was measured by high-performance liquid chromatography (HPLC). The method employed on the: CN RP column (250 mm × 4.6 mm, 5 µm particle size) from Macherey-Nagel (Germany), as the stationary phase. The mixture of acetonitrile and water (40:60) containing 100 mM tetra methyl ammonium chloride (pH = 4.2) was used as a mobile phase. Propranolol was used as an internal standard and injection volume was 100 µL. All the chromatograms were recorded at 204 nm with mobile flow rate of 1 mL/min. The release profile was evaluated as the cumulative percentage of clindamycin released in the medium.

### 3.4. Differential Scanning Calorimetry Analysis

Samples were ground and approximately 10 mg from each sample was analyzed. To record DSC (differential scanning calorimetry) thermograms, matrices were sealed into DSC aluminum sample pans. Thermograms were recorded on a differential scanning calorimeter (Mettler ToleDo, Star system, USA) using an empty pan as a reference. Scans were recorded with a rate of 10˚C/minute between 20˚C and 100˚C. All DSC data analysis was performed using STAR SW version 9.10.

### 3.5. Animals

A total of 30 New Zealand white rabbits of either sex (2-2.5 kg) were purchased from the Pasteur institute (Tehran, Iran). All animal procedures were performed in accordance with the Association for Research in Vision and Ophthalmology (ARVO) statement on the use of animals in ophthalmological research. The animals were divided into two groups as follows:

Group I: received unloaded intravitreal lipid implants (n = 15 rabbits);
Group II: received unloaded intravitreal PLA implants (n = 15 rabbits);

The left eyes in each group were maintained untreated as a control.

The rabbits were adequately anesthetized with I.M. xylazine (10 mg/kg) and ketamine (50 mg/kg), followed by topical anesthesia with tetracaine (1%). The eyes were cleaned with povidone-iodine (5%) as a pre-operative antiseptic and draped with a sterile cloth. For inserting the implants, under sterile conditions, a 23-gauge cannula was inserted 1 mm posterior to the limbus. Next, implants were introduced through this cannula inside the virtues cavity. The sclera was sutured with 8-0 acryl. All the rabbits recovered without any complications. Chloramphenicol and betamethasone topical drops were applied after the procedure and every six hours during the three following days.

### 3.6. Biocompatibility

#### 3.6.1. Clinical Observations

Indirect ophthalmoscopy was done at baseline (before experiment on day one), immediately after administration of implant and periodically during the experiment. In addition, any clinical signs of inflammation, conjunctivitis and discharge were noticed.

#### 3.6.2. Electrophysiological Studies

After anesthetization with I.M. xylazine (10 mg/kg) and ketamine (50 mg/kg), pupil dilatation was done with 1% tropicamide and 2.5% phenylephrine. Next, rabbits were dark-adapted for 20 minutes. The scotopic electroretinograms (Metrovision Win 7000, Prefenchies, France) were recorded before and one, two, four and eight weeks after implantation. Changes in scotopic b-wave amplitudes before and after intravitreal implantation were analyzed with the SPSS 14.0 software. Differences were considered statistically significant if P < 0.05. Non-parametric statistics throughout Wilcoxon Signed-Rank test was applied to determine differences between case and control groups.

#### 3.6.3. Histological Studies

For histological examinations, at each time point, the eyes were dissected under a microscope. The eyes were fixed in formalin 10% immediately after enuculation. After tissue processing, the samples were embedded in paraffin and sectioned in 4 µm thick sections. The sections were stained with hematoxylin-eosin, examined under a light microscope and photographed with a digital camera.

## 4. Results

### 4.1. In vitro Characteristics

The manufactured CLP-loaded matrices had a well-defined cylindrical geometry. The mean drug content was calculated as 550 µg ± 24 µg. The small size of these matrices let them to be injectable via a trocar.

#### 4.1.1. In vitro Release Test

**Figure 2.**
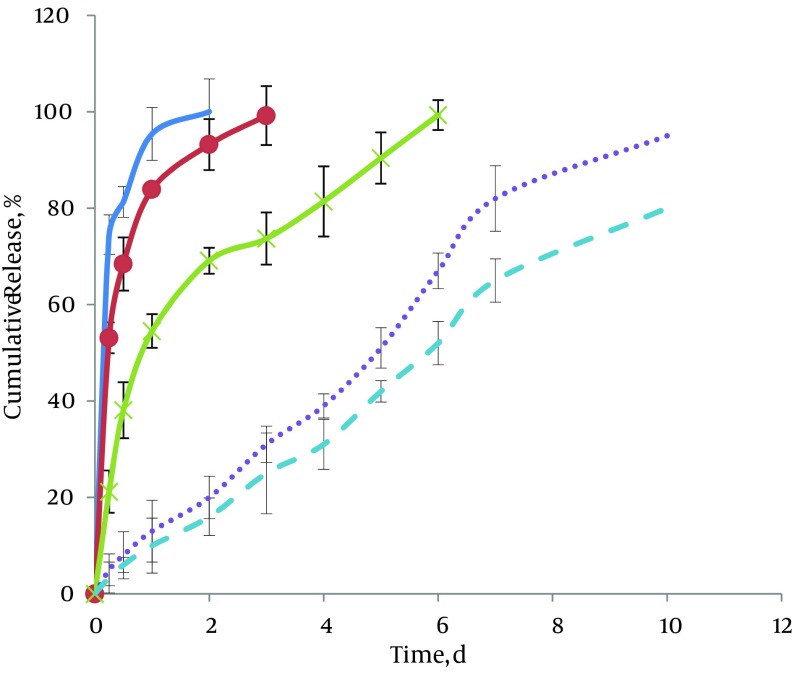
Comparison of CLP *in vitro* Release From Matrices Containing 50% GTP (Solid Line), 60% GTP (o), 80% (x), 90% (…..) and 95% (----) GTP

Figure 2 represents the plots of cumulative percentage released vs. time for matrix-embedded controlled release extrudates of CLP. Comparisons indicate the effects of different ratios of GTP to CLP on release profiles. As shown in these figures, with 50% and 60% of GTP, two-phase cumulative release profiles were observed. The first phase, i.e. burst release phase, was obtained after 6 hours of incubation. By using higher GTP ratios (80-95%), decreases in the first part of the diagram were seen. The cumulative release percentage for this part reduced from 74.5% ± 4% in case of 50% GTP to 53.1% ± 4 and 21.2% ± 3 for 60% and 80% GTP, respectively. Results showed that when the GTP part of the matrices increased to 90% and 95%, the initial burst effect decreased due to the presence of hydrophobic material, which depress the initial penetration of water into the system (5% ± 0.8% and 3% ± 0.5, respectively). The second portion of the curves is a sustained release phase. Duration of this part of the curves also varied according to GTP ratios. As shown in Figure 2, for 50% GTP, drug release was complete after two days. With increasing GTP ratios, more controlled-release patterns were obtained. Therefore, in case of 95% GTP, this duration, increased up to ten days.

#### 4.1.2. DSC

**Figure 3.**
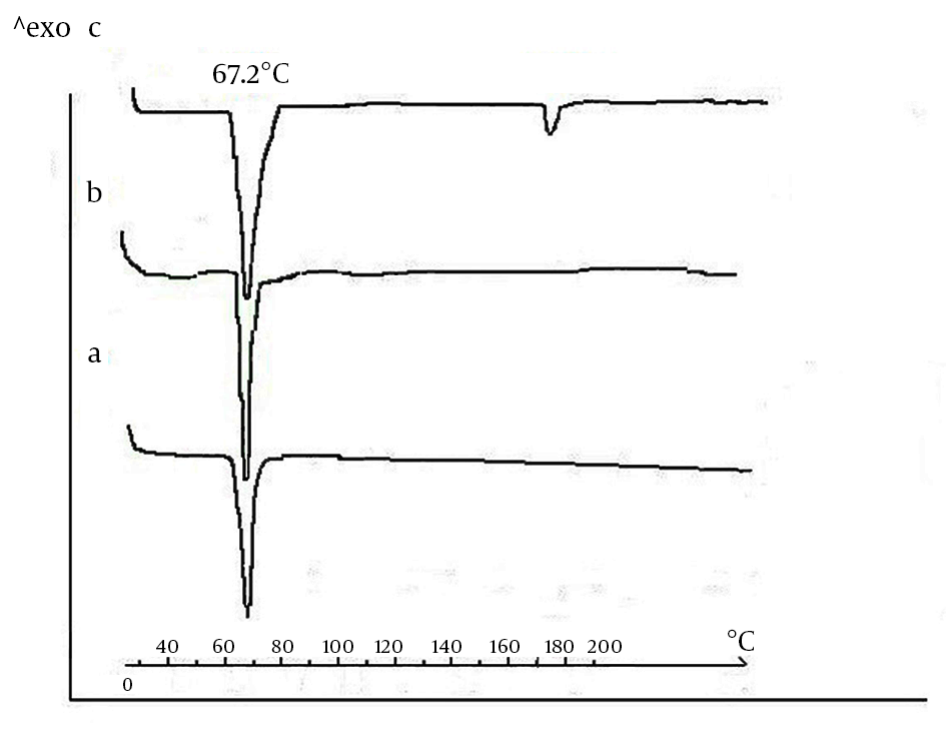
DSC Images of GTP A) Untreated; B) Sterilized with heat; C) LP-loaded implant. The thermograms were recorded at a heating rate of 100˚C/min.

It is known that polymorphic transformations may occure during the processing of lipids. Therefore, it seems necessary to investigate if the manufacturing protocol affects the modification of the triglyceride components. For this reason, DSC thermograms were recorded for the GTP bulk sample after heat sterilization and after compression (Figure 3). GTP bulk samples (Figure 3A) showed a single endothermic transition at 67.2˚C that stemmed from the melting of the crystalline ß-modifications. Scans obtained for GTP matrices were identical with those obtained for the bulk material. This showed that no polymorphic transformations occurred during sterilization and compression and that the stable crystalline ß-modification was largely maintained (Figures 3B and 3C). Thermograms of CLP loaded-implants (Figure 3C) showed two endothermic peaks corresponding to the melting endotherm of the two substances. While the location of the triglyceride melting endotherm remained constant at 67.2˚C, the drug peak appeared at 172.4˚C, corresponding to the CLP melting point. From these observations, we concluded that GTP crystallized again in the stable ß-modification. Furthermore, one can assume that CLP and triglyceride seem to coexist in separate phases.

### 4.2. Toxicology Studies

**Figure 4.**
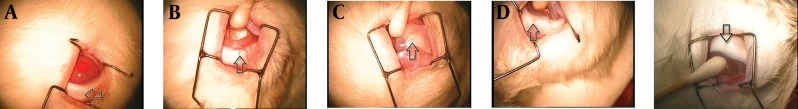
Clinical Photographs of the Rabbits’ Implantation Sites A) Before implantation; B) immediately after implantation; C) and two weeks; D) one month; E) and two months after implantation.

**Figure 5.**
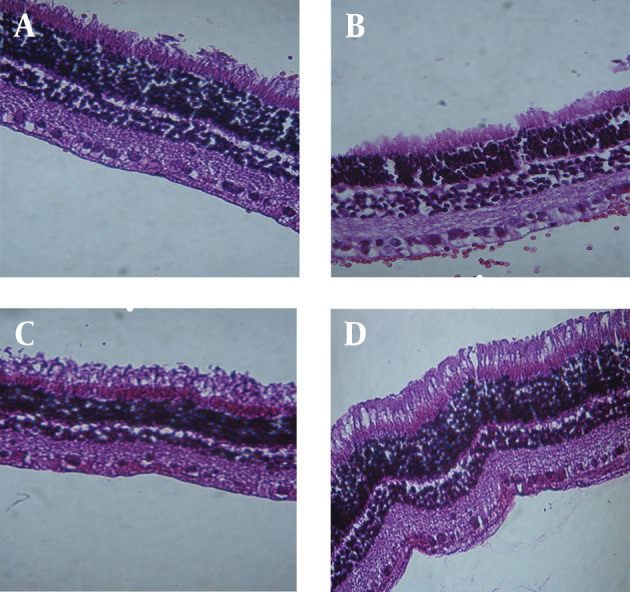
Histological Section of Rabbit Retina Showing a Normal Architecture A) At 3th day; B) two weeks; C) four weeks D) and eight weeks post-implantation

Biocompatibility study of GTP matrices was performed comparing them to PLA rods; a biocompatible polymer commonly used in fabrication of ocular sustained release dosage forms. Periodic clinical observations indicated no signs of inflammatory reactions (including hazy vitreous, fibrosis, membranous opacity, hemorrhage, retinal folding, and retinal thickness) of retinochoroidal tissue in response to the presence of the implant. Microscopically observations showed no significant inflammatory response at implantation sites; Figure 4 shows *in vivo* observation of the implants after surgical implantation into the vitreous of rabbit eyes. As clearly indicated, no clinical inflammation was seen at weeks two, four and eight after implantation. Histological studies showed that there was no evidence of retinal abnormalities near the implantation sites 8 weeks post-implantation (Figure 5); the images show, the retina maintained its structure. There was no evidence of decrease in ganglionic cells in both outer and inner nuclear cells and also no sign of change in retina thickness.

Indirect ophthalmoscopy demonstrated no abnormal findings in retinas during the observation period. Scotopic b-wave amplitude ratios before and after implantation were not significantly different (P > 0.05). In addition, there was no significant difference in these parameters between GTP and PLA implants (P > 0.05).

## 5. Discussion

Biocompatible and biodegradable excipients have been widely investigated for designing ocular drug delivery systems for the elimination of potential toxicity of current delivery systems (1). Natural materials derived from biological systems including protein, lipids, and polysaccharides are considered as potential biocompatible and biodegradable materials. The hypothesis was based on the fact that due to their natural sources they may possess low toxicity and potentially favorable pharmacokinetics in the body. Physiological triglycerides have repeatedly been indicated as promising by *in vitro* considerations as an alternative to synthetic polymers. These materials are formulated in to microparticles and cylindrical matrices loaded with therapeutically active ingredients (3-6). In the direction of using lipids as carriers, the biocompatibility of triglycerides and monoglycerides was investigated for limited parts of the body.

In the present study, GTP was selected as a matric former. The major advantages of GTP compared to polymers are its favorable processability, low swelling ratio in aqueous media and lower melting point (about 40˚C), which allows for lower fabrication temperatures (5, 8). There are some reports of successful utilizations of GTP for the fabrication of sustained release systems. Reithmeier et al. reported the use of lipidic microparticles from GTP for the parenteral administration of peptides. The systems showed good biocompatibility in mice and had promising drug release profiles (9). Similarly, somatostatin was encapsulated into GTP-based microparticles (11). The devices, demonstrated *in vitro* drug release over ten days. Vogelhuber et al. prepared lipid-based cylindrical matrices using GTP to incorporate a small molecular weight fluorescent model substance, pyranine, which subsequently showed a sustained release for more than 120 days (6). Koening et al. considered GTP matrices as potential carriers for controlled release of a brain-derived neurotrophic factor. *In vivo* evaluation in the rat brain, revealed its biocompatibility compared to reference cylinders (8).

To our best knowledge, by far, there was no study on the preparation and post *in vivo* investigations of lipid implants for intraocular purposes. It was the intention of this study to develop an implantable delivery system for CLP, based on a triglyceride of GTP, which may allow drug release in a linear mode during the 5-6 weeks of treatment. CLP is an antitoxoplasmosis agent where its intravitreal injection especially in case of unresponsive retinotoxoplasmosis, development of serious side effects with systemic therapeutics and also in the case of pregnancy is becoming of high interest. However, its short half-life makes it a suitable candidate for incorporation in sustained release delivery systems (12, 13). The first part of this research described the manufacturing strategy. Aseptic fabrication is used for production of the sterile lipid-based delivery system. Here, in this study, sterilization of GTP was performed by heating at 120˚C. The only problem related to this method can be the concern for changes in the crystalline structure of the lipids. In this study, any modification in GTP during tempering was eliminated using a method described by Appel et al. (11). DSC approved keeping the primary crystalline structure of GTP during sterilization and also the manufacturing process.

Most manufacturing strategies for lipid cylinders have so far relied on the simplicity of direct compression of a powder mixture of lipid and active ingredients which could not guarantee homogenous mixtures of compounds. The manufacturing procedure described in this paper, allows for easy handling of small batch sizes for the preparation of a homogeneous lipid implant loaded with either hydrophilic or lipophilic drugs. As shown by Figure 1, the first step aimed to obtain a homogeneous dispersion of CLP and GTP. Due to different solubility characteristics, a homogenous mixture was formed via formation of W/O emulsion. This mixture was obtained by a short application of ultrasound after the addition of the CLP solution to the lipid solution. The formulation was frozen in liquid nitrogen to induce the precipitation of dissolved lipid on dispersed CLP microparticles, resulting in a very fine and homogeneous distribution of the drug within the lipid powder mixture. After applying the vacuum, a solid powder of the mixture was obtained. Continuous *in vitro* release profiles of implants confirmed the formation of homogenous matrices.

The homogenous mixture was placed under extrusion to obtain solid micro-implants. In this study, the lipid is not molten and only treated below its melting range to obtain a semi-solid mass. By applying appropriate pressure, the raw materials passed through a die and micro-implants with desirable shape and diameter were obtained. Here, by using a die with a 0.41 mm diameter, the miniaturized implant, which can easily be inserted inside the eye, through a trocar, was made. In *in vitro* release tests, the formulations with 95% lipid content, showed more prolonged release profiles. Although, it showed a relatively good retardation of CLP yet it was not adequately sustained for the delivery of CLP during the treatment of retinotoxoplasmosis (which was reported for about 4-5 weeks) (14).

In the second part of this approach, biocompatibility of fabricated implants was studied for investigation of its potential as a carrier in the intraocular sustained release dosage forms. Since drug loading implants did not represent an appropriate system for delivery of CLP, only blank implants underwent *in vivo* bioavailability test. In this regard, un-loaded GTP implants were inserted into the rabbits eyes and clinical studies were done and compared to blank PLA which in previous studies (3), were reported as a safe material for fabrications of intraocular implants. These investigations did not show any toxicity related to GTP. In comparison with PLA, there was no significant difference in the clinical parameters, during the two-month duration of the studies.

In our study, we prepared natural lipid micro-implants in the size range suitable for intraocular injection by a simple compression method. It was shown that the developed extrusion protocol neither affects the modification of the lipids nor the stability of the incorporated model drug. Moreover, the drug could be homogeneously distributed within the lipid matrix. The best results were obtained with a drug loading of 5%. The drug was released *in vitro* almost continuously over approximately ten days. An *in vivo* evaluation of these matrices was undertaken in order to evaluate their applicability as potential carriers for loading a wide range of therapeutics for the treatment of retinochoroidal diseases. The results were promising in regards to good biocompatibility of GTP with retinal tissue. In addition to good tolerance, easy manufacturing and scaling up of the developed implants indicate that they are good alternatives for synthetic polymers for the ocular sustained release drug delivery systems.
